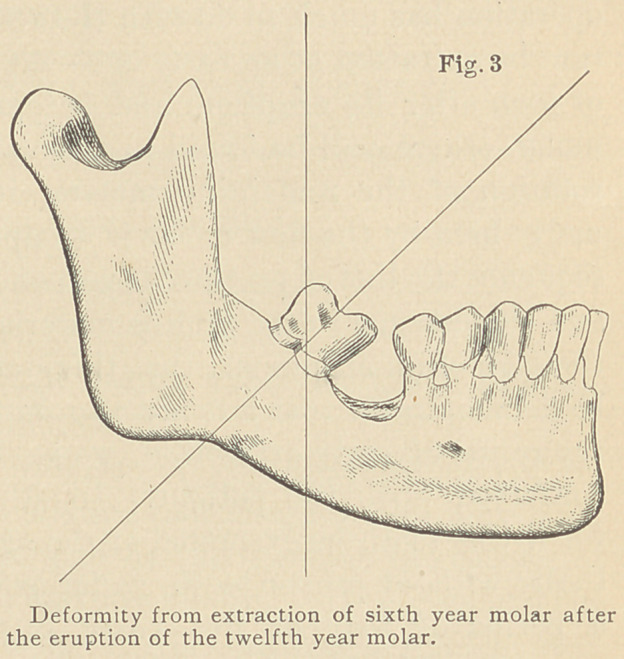# The Extraction of Molars

**Published:** 1888-02

**Authors:** G. W. Weld


### THE EXTRACTION OF THE FIRST OR SIXTH YEAR MOLARS.

Read before the Central Dental Association of Northern New Jersey, December 19, 1887.

BY DR. G. W. WELD.

A paper entitled "The Significance of the Natural Form and Arrangement of the Dental Arches of Man, with a Consideration of the Changes Which Occur as a Result of Their Artificial Derangement by Filling or by the Extraction of the Teeth," by Dr. I. B. Davenport, of Paris, was read before the New York Odontological Society, on Tuesday evening, April 12, 1887.

The paper above referred to illustrates, more than any study which the writer has ever considered, the injury which is sometimes inflicted on the dental arches of man by the premature extraction of this tooth. Indeed, from an examination of the models and drawings that accompanied the paper in question, one is almost forced to accept its conclusions, viz., that herewith those practitioners of dentistry who have been in the habit of extracting this tooth for the purpose of regulating the teeth have only made themselves conspicuous in error, and in the end produced an irregularity more difficult of correction than that which previously existed.

The importance of this tooth in completing the natural form and arrangement of the dental arches is fully illustrated by a consideration of its absence or loss in connection with the laws of the equilibrium of pressures. These laws were virtually embraced, if not expressed, in Dr. Davenport's theses, and it is concerning these laws—a repetition of the same sounds, a re-echo, so to speak—that I invite your attention this evening.

In civil architecture, as, for instance, in the construction of an arch, mathematicians have endeavored to render the subject easy of comprehension by introducing certain pre-supposed conditions. Thus, in treatises on the theory of the arch, the structure is regarded as consisting of a course of arch-stones resting on abutments and carrying a load which is supposed to press only downwards upon the arch-stones; and also that in such cases cohesion and friction are entirely lost sight of, and the investigation is conducted as if the stones could slide freely on each other. For example, if the line of pressure of one stone against another be across their mutual surfaces perpendicularly, there is no tendency to slide; and if this condition be adhered to throughout the whole structure, there must result complete stability. But if under any circumstances the line of pressure should cross the mutual surfaces of the arch-stones obliquely, the tendency to slide must be resisted only by cohesion, and the stability of the arch would at once be impaired. It is now, I believe, generally admitted that the line of pressure on an arch is perpendicular and at right angles to the horizontal line of base.

A simple stone arch, similar to that represented in the above diagram, is self-supporting only when the two haunches A. A. are secured by an iron tie rod O. O., unless they (the haunches) are sufficiently heavy to withstand the thrusts. The line of pressure is vertical at the centre of the keystone E., but becomes more and more oblique as the stones B. B. are approached, the tendency being to cause it to kick out at the haunches A. A. This is when the arch sustains no weight more than the stone blocks composing the arch. In setting the arch the stones, A. A. and B. B. would hold themselves in position without cement by natural friction of surfaces, if the joint M. did not make an angle with the horizontal line N. (at the springing of the arch) of more than twenty degrees; stones C. C. and D. D. would slide off and could only be held in position by the insertion of the keystone E.

When the arch sustains a load equally disposed above it, the thrust is, of course, perpendicular at the keystone, and also at all other points until it strikes the arch itself, where the tendency is to slide off at a tangent, as indicated in the lines F. G. H., causing the abutment to kick out, if it is not strong enough to withstand the thrusts. In case sufficient material is piled above the arch (supposing the lateral supports are sufficiently strong) it would undoubtedly first crush the stones C. C. If there was more weight at the sides than over the keystone, the arch would fail at the central point by the crumbling of the key.

A well-constructed stone arch, properly cemented, becomes practically a single stone, and fails at its weakest points when overloaded, these points being, as above mentioned, at C. C.

Let us now consider the dental arch—an arch composed of teeth instead of stones—and endeavor to point out the effect which follows the removal of the first molar.

* In an article recently published in the "American System of Dentistry," entitled the Geometrical and Mechanical Laws of the Articulation of the Human Teeth, Dr. Bonwill very truly says that in order to comprehend what constitutes true articulation of artificial teeth, it becomes necessary to study the anatomy of the human jaw and its functions.

"The study of this one part of the head and jaws shows one of the most striking designs of an architect; and when studied it will be seen that every part of our frame is made by a positive law and to subserve definite purposes, such a law being in consonance with geometry, physics and mechanics. We must see the true use or function of the jaw and the teeth, and the food destined for us and

---

* "The Geometrical and Mechanical Laws of the Articulation of the Human Teeth. The Anatomical Articulator," by W. G. A. Bonwill, D. D. S.

how it should be communicated. There is no chance work about it. Law and order pervade everywhere."

Fig. 1

In the simple arch we found that the keystone occupied the most central position. In the complete dental arch we shall find the first molar to be the most centrally located, so that it may be properly called the keystone. We shall also find in the complete dental arch that the law of the equilibrium of pressures is as fully applicable as in the finished architectural structure of a simple or complicated arch. In the simple arch we find the line of pressure to be perpendicular on the keystone, and that whenever the line of pressure of one stone against the other was across their mutual surfaces perpendicularly, there was no tendency to slide, and that there was complete stability. In the complete dental arch the line of pressure or thrust is also perpendicular to the axis of the roots of the teeth.

Fig. 2

In the incomplete dental arch, or after the removal of the first molar, particularly of the lower jaw, the natural form and arrangement of the teeth is interfered with and impaired. For example, when the first inferior molar is removed (see Fig. 2) the superior and first molar being left in the arch, we find that the posterior edge of the first superior molar comes in contact with the anterior edge of the second inferior molar, causing a line of pressure or thrust that is not perpendicular to the axis of the roots of the second and third molars. Under such conditions, with a natural tendency of the second and third molars to slip forward, one of two things must happen; either the foundation of the setting must be sufficiently strong to resist rotation, or disarticulation must occur. Uufortunately, the foundation or setting in the absence of the first molar is materially weakened, so that when the line of pressure of one tooth against another is not in harmony with their mutual surfaces, or when the pressure or thrust is directed against the axis of the roots of the remaining molars, their crowns are tipped forward, the axis of their roots is changed, disarticulation occurs and the once beautifully formed arch becomes a wreck. The condition is similar to that represented in an arch that is not self-supporting, *i. e.*, when the haunches are insecured, or are not sufficiently heavy to withstand the thrusts.

Fig. 3

Deformity from extraction of sixth year molar after the eruption of the twelfth year molar.

*Dr. Guilford, in a recent article on Orthodontia, referring to extraction for irregularity, states that he is in the habit of selecting "the one nearest and posterior to the one out of position." This rule, which is to be commended, would necessarily at times point to the extraction of the sixth year molar; but, nevertheless, the principles involved in the preservation of the arch are not changed, and the selection of any other tooth for extraction back of the cuspids must, as a rule, do far less injury to the setting.

It has been argued that this tooth, being the least permanent of all the permanent teeth, should generally be selected as the one to be sacrificed. But the advocates of this theory seem to forget that this tooth works harder, and endures more neglect and adverse conditions than any other tooth in the head. Think of a tooth living in the mouth during the trying years of childhood, and subjected constantly to all sorts of unfavorable conditions, such, for instance, as the presence of decaying teeth, a lack of cleanliness, a vitiated saliva, acid eructations from the stomach, neglect of parents, and in many cases indifference on the part of the dentist regarding its importance. No wonder that statistics show it to be less permanent than the so-called permanent teeth. But could the bicuspids or the second molars, for instance, run the same gauntlet and suffer less? Let the practitioners who uphold this theory first show that the first molar does not suffer and endure more than

---

* Orthodontia, by S. H. Guilford, A. M., D. D. S. American System of Dentistry, Vol. II, p. 328.

the other teeth before they question its constitution and draw the line in favor of extraction.

But we are told that the age at which the patient undergoes the operation has much to do with the result, *i. e.*, if the molar in question be extracted at an early age—say in the seventh or eighth year, or soon after its eruption, and before the eruption of the second molar—there will be found in after years a translation instead of a rotation of the remaining molars; in other words, the space formerly held by the first molar is occupied by the second, and in consequence the line of pressure is perpendicular to the axis of the roots, and the damage to the arch is materially lessened. Admitting, for the sake of an argument, that this statement is true, and that it can be demonstrated that a modified rotation or complete translation is the result of early extraction, we are led to inquire what benefit is conferred upon the patient. In either case there is a deprivation, for if this tooth be extracted at the age of seven the patient is virtually without proper teeth, so far as mastication is concerned, for a period of four or five years, or until the second molars are erupted. The four first molars make up a whole set of teeth for a young person. Their absence from the mouth during childhood involves the loss of nutrition; it presupposes indigestion, dyspepsia, the lack of proper food assimilation, and a deterioration in health. Dr. Carl Heitzmann spoke true words indeed when he said that "the dentist who would try to prevent future disease by extracting a tooth plays Providence, and we all know this is a dangerous play."

In conclusion it may be said of the sixth year molar:

I. Its title to longevity can only be questioned under neglect and abuse.

II. It is the keystone molar; with it the integrity of the arch is preserved; without it the usefulness of the arch is impaired, if not destroyed.

III. Its extraction at an early period signifies a loss of masticating surface that is absolutely detrimental to the health and comfort of the patient in early life.

IV. In view of the prominent position it occupies in the arch and its relation and influence as a just poise or balance in the distribution of the varied strains incident to mastication, its extraction can only be considered a physiological mistake.

## Figures and Tables

**Figure f1:**
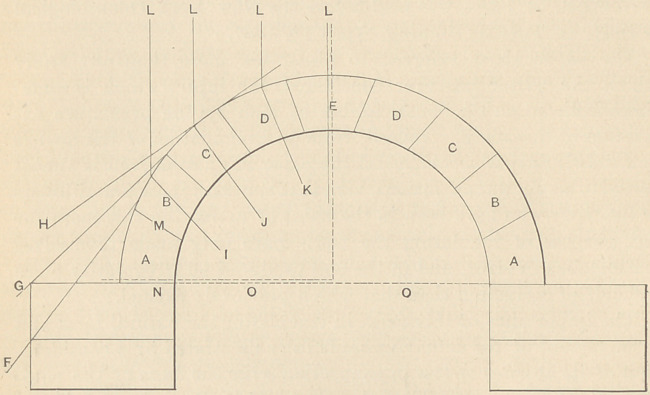


**Fig. 1 f2:**
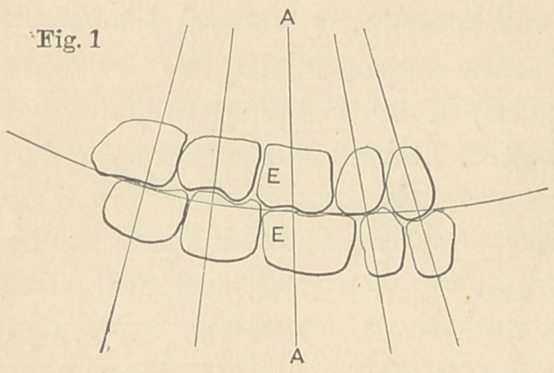


**Fig. 2 f3:**
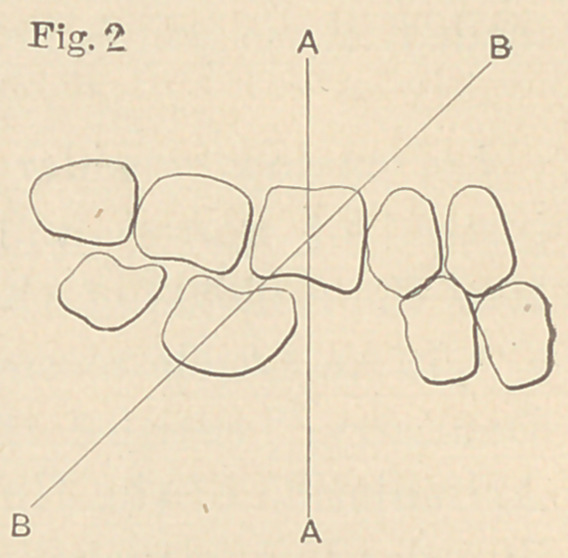


**Fig. 3 f4:**